# Early-Age-Onset Colorectal Cancer in Canada: Evidence, Issues and Calls to Action

**DOI:** 10.3390/curroncol29050256

**Published:** 2022-04-29

**Authors:** Mary A. De Vera, Sharlene Gill, Shady Ashamalla, Dan Schiller, Darren R. Brenner, Clarence Wong, Petra Wildgoose, Mary Jane Esplen, Christopher Lieu, Roslyn Fitzpatrick, Dylan E. O’Sullivan, Filomena Servidio-Italiano

**Affiliations:** 1 Faculty of Pharmaceutical Sciences, University of British Columbia, Vancouver, BC V6T 1Z3, Canada; mdevera@mail.ubc.ca; 2 Division of Medical Oncology, BC Cancer, University of British Columbia, Vancouver, BC V5Z 4E6, Canada; 3 Department of Surgery, University of Toronto, Toronto, ON M5T 1P5, Canada; shady.ashamalla@sunnybrook.ca; 4 Sunnybrook Health Sciences Centre, Toronto, ON M4N 3M5, Canada; petra.wildgoose@sunnybrook.ca; 5 Department of Surgery, University of Alberta, Edmonton, AB T5H 3V9, Canada; ds9@ualberta.ca; 6 Department of Community Health Sciences, University of Calgary, Calgary, AB T2N 4N1, Canada; darren.brenner@ucalgary.ca (D.R.B.); dylan.osullivan@ucalgary.ca (D.E.O.); 7 Department of Oncology, University of Calgary, Calgary, AB T2N 4N1, Canada; 8 Department of Cancer Epidemiology and Prevention Research, Alberta Health Services, Calgary, AB T2N 4N1, Canada; 9 Forzani & MacPhail Colon Cancer Screening Centre, Alberta Health Services, Calgary, AB T2N 4N1, Canada; 10 Division of Gastroenterology, University of Alberta, Edmonton, AB T6G 2X8, Canada; ckw3@ualberta.ca; 11 Department of Psychiatry, University of Toronto, Toronto, ON M5T 1P5, Canada; maryjane.esplen@utoronto.ca; 12 Division of Medical Oncology, Gastrointestinal Medical Oncology, University of Colorado Anschutz Medical Campus, Aurora, CO 80045, USA; christopher.lieu@cuanschutz.edu; 13 Patient Advocate, Thornhill, ON L3T 3H1, Canada; rozfitzpatrick@yahoo.ca; 14 Colorectal Cancer Resource & Action Network, 700-2 Bloor Street West, Toronto, ON M4W 3E2, Canada; filomena.s@ccran.org

**Keywords:** early-age-onset colorectal cancer, EAO-CRC, EO-CRC, colorectal cancer

## Abstract

The inaugural Early-Age-Onset Colorectal Cancer Symposium was convened in June 2021 to discuss the implications of rapidly rising rates of early-age-onset colorectal cancer (EAO-CRC) in Canadians under the age of 50 and the impactful outcomes associated with this disease. While the incidence of CRC is declining in people over the age of 50 in Canada and other developed countries worldwide, it is significantly rising in younger people. Canadians born after 1980 are 2 to 2.5 times more likely to be diagnosed with CRC before the age of 50 than previous generations at the same age. While the etiology of EAO-CRC is largely unknown, its characteristics differ in many key ways from CRC diagnosed in older people and warrant a specific approach to risk factor identification, early detection and treatment. Participants of the symposium offered directions for research and clinical practice, and developed actionable recommendations to address the unique needs of these individuals diagnosed with EAO-CRC. Calls for action emerging from the symposium included: increased awareness of EAO-CRC among public and primary care practitioners; promotion of early detection programs in younger populations; and the continuation of research to identify unique risk factor profiles, tumour characteristics and treatment models that can inform tailored approaches to the management of EAO-CRC.

## 1. Introduction

In light of the rapidly rising rates of early-age-onset colorectal cancer (EAO-CRC) among Canadians under the age of 50 and the impactful outcomes associated with this disease, the Colorectal Cancer Resource & Action Network (CCRAN) convened the inaugural Early-Age-Onset Colorectal Cancer Symposium in June 2021. This virtual symposium brought together a cross-section of patients, clinicians and researchers to discuss the implications of the current evidence on EAO-CRC and to offer directions for research and clinical practice. This report summarizes the highlights of the symposium and recommendations for action. 

### 1.1. Symposium Objectives

The objectives of the symposium were:

- To summarize evidence and educate on the issues related to EAO-CRC;
- To prioritize responses to address issues related to EAO-CRC in Canada; and
- To influence practice change amongst primary care physicians or providers to support the early diagnosis of EAO-CRC.

### 1.2. Symposium Organization

The symposium was organized under the auspices of CCRAN, a colorectal cancer (CRC) patient and caregiver support, education and advocacy network in Canada. It was co-chaired by Dr. Shady Ashamalla, Head, Colorectal Cancer Surgical Oncology, Sunnybrook Health Sciences Centre, Toronto and Dr. Sharlene Gill, Medical Oncologist, BC Cancer Agency, Vancouver. Facilitation was provided by Ms. Anne Marie Wright of Elements Strategy Inc. and an Expert Steering Committee, consisting of the authors of this paper, provided direction on the symposium objectives, agenda, speakers and invited participants. 

### 1.3. Participants

A total of 170 people registered for the symposium. Approximately 80 attended on the day of the event and other registrants accessed the recorded session online afterwards. Of the participants, 42% identified as patients or belonged to a patient group, and 33% were healthcare professionals or researchers.

### 1.4. Agenda

Due to COVID-19 pandemic restrictions, the symposium was held by online videoconference. Audience members interacted with presenters by asking questions and providing comments online. The symposium agenda is shown in Table 1.

## 2. Evidence Review

### 2.1. Incidence

Colorectal cancer is the third most common cancer in Canada with approximately 24,800 cases projected to have been diagnosed in 2021 [1]. Although the incidence of CRC among adults under the age of 50 represents approximately 8% of CRC cases in Canada [1], recent evidence shows that the incidence of EAO-CRC in younger people is rising rapidly in Canada and in other developed countries worldwide [2,3,4,5]. 

**Figure 1:**
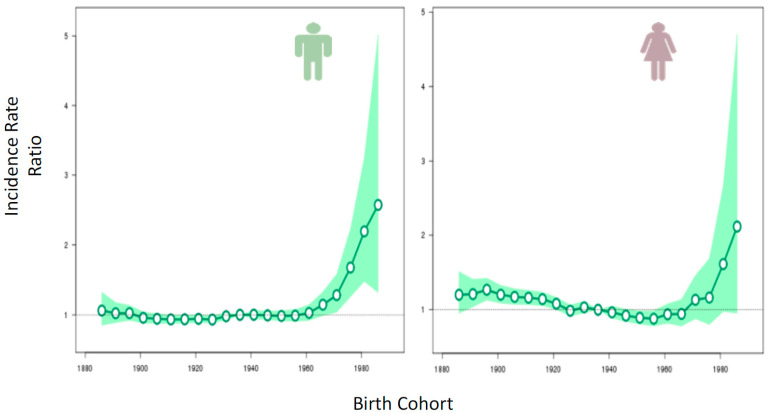
Risk of colorectal cancer by birth cohort, Canada 1888–1988. Reprinted with the permission from Ref. [6] 2019.

As shown in Figure 1, the population born since 1980 is 2 to 2.5 times more likely to be diagnosed with CRC before age 50 than were previous generations at the same age. 

**Figure 2:**
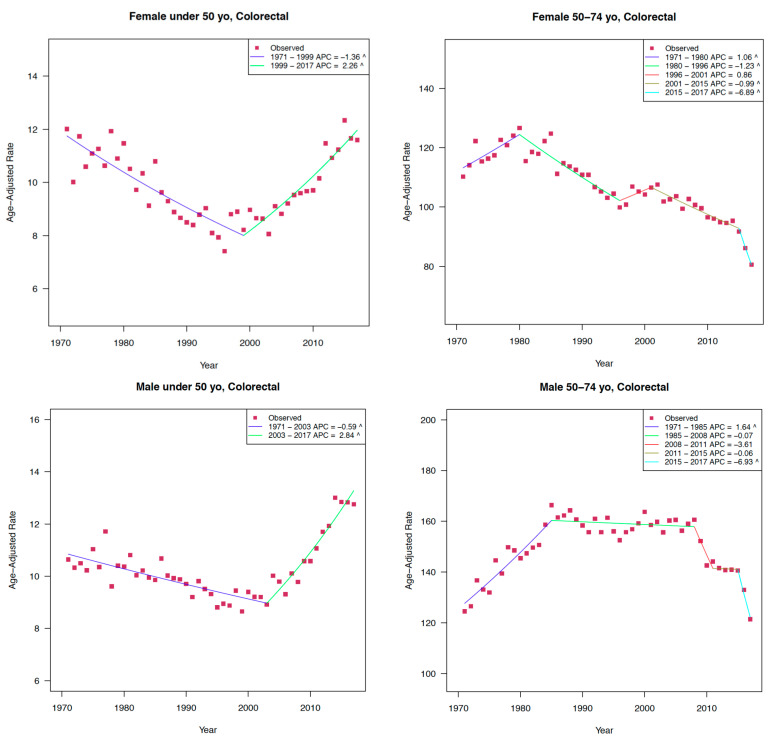
Colorectal cancer incidence trends by age group, Canada 1970–2017. Reprinted with the permission from Ref. [2] 2021.

This pattern is even more alarming set against the backdrop of a decreasing incidence of CRC among the 50–74 age group shown in Figure 2. The declining incidence of CRC in people over 50 has been attributed to multiple factors such as organized screening programs, reduced smoking rates, and the consumption of anti-inflammatory medications [7]. 

Similar patterns have been documented in several other high-income countries [8], leading to suggestions that this younger population may be exposed to environmental or lifestyle factors that predispose them to develop CRC earlier in life [3,9,10,11]. In Canada, there is some evidence that the incidence of EAO-CRC varies by socioeconomic status, with greater increases in incidence among individuals 20–44 residing in lower income neighbourhoods, as well as greater increases among individuals 45–49 residing in higher income neighbourhoods [12]. In addition, there is some evidence that EAO-CRC is more common among individuals living in urban areas. 

### 2.2. Tumour Characteristics

Younger patients tend to be diagnosed at later stages of disease [13,14,15]. Approximately 60% of younger persons are diagnosed at Stage III or IV compared to approximately 50% for patients over the age of 50 [16]. 

Younger patients are more likely than their older counterparts to develop tumours of the rectum (approximately 25% versus 15%) [17,18], which are more aggressive and more difficult to treat than colon cancers. Treatment of rectal cancer typically includes surgery, radiation therapy and chemotherapy which can have long-term impacts on sexuality, fertility and pregnancy, which are of importance to younger patients [8]. Because recurrence is common, therapeutic options can be exhausted over time.

Another important observation is that CRCs in younger patients are more likely to develop on the left side. Tumour sidedness predicts response to anti-epidermal growth factor receptor (EGFR) therapies. For example, emerging evidence suggests that patients with left-sided colon cancers benefit from cetuximab or panitumumab as first-line therapy for metastatic disease [19].

### 2.3. Genetics and Genomics

Genetic transmission is an important risk factor for EAO-CRC. Studies have shown that 25–35% of EAO-CRC patients have a first-degree relative who was diagnosed with CRC [19]. 

Compared to older patients, people born after 1970 tend to show a distinctive set of risk factors at the molecular level, including [20]:

- More microsatellite instability (MSI-high);
- Fewer BRAF mutations and mutations in MAPK gene pathways;
- More chromosomal instability;
- More copy number alterations;
- Greater long interspersed nuclear element-1 (LINE-1) DNA hypomethylation;
- Less DNA hypermethylation in promoter regions;
- More MSI/immune or canonical APC/B-catenin subtypes;
- Less metabolic or mesenchymal subtypes.

Because the biology of tumours in younger and older patients differs, understanding how genomes and exosomes interact in the development of cancer is a key field of ongoing research. 

Another field of exploration is the characterization of “mutational signatures” consisting of combinations of genomic alterations, which could be consistent with novel exposures that may be driving the significant increases in risk for younger people. This is particularly important for exposures that are difficult to study or would take a long time to establish in traditional epidemiologic study designs (prospective cohort or case-control). 

### 2.4. Primary Prevention: Risk Factor Identification

Because it is unexpected at a younger age, many patients and primary care providers lack an awareness of the link between known risk factors and EAO-CRC. 

A recent review of the international literature showed strong evidence for heredity as a more important risk factor for younger populations than for those over 50 [2]. Individuals who have a first-degree relative with a family history of CRC have 2-fold increased risk of CRC in general [21], but a 4-fold increased risk of EAO-CRC compared to individuals without a family history of CRC [17]. Among younger patients with a family history of CRC, about 20% are affected by a germline mutation with a detectable hereditary condition, such as [20]:

- Lynch syndrome, also called hereditary nonpolyposis colorectal cancer (HNPCC);
- Familial adenomatous polyposis (FAP);
- Attenuated familial adenomatous polyposis (AFAP), a subtype of FAP;
- Gardner syndrome, a subtype of FAP;
- Juvenile polyposis syndrome (JPS);
- Muir–Torre syndrome, a subtype of Lynch syndrome;
- MYH-associated polyposis (MAP);
- Peutz–Jeghers syndrome (PJS);
- Turcot syndrome, a subtype of FAP and Lynch syndrome.

Despite this knowledge, family history is often overlooked by both patients and primary care providers. While nearly 28% of respondents in the US Colorectal Cancer Alliance’s “Never Too Young” survey of 885 EAO-CRC patients indicated they have a family history of CRC, only 52% were aware that this increased their risk for CRC [15]. Similar findings were reported in a Canadian study, where fewer than half of patients correctly assessed their CRC risk based on family history [22].

In their review, O’Sullivan et al. evaluated the scientific evidence linking risk factors to the development of CRC in adults under 50 [2]:

- Moderate evidence was found for male sex, obesity and alcohol consumption;
- Suggestive evidence was found for Caucasian ethnicity and cigarette smoking;
- Emerging evidence was found for the role of other risk factors including:
  o Comorbidities: hyperlipidemia, diabetes, ulcerative colitis, hypertension, metabolic syndrome, and chronic kidney disease;
  o Lifestyle factors: sedentary behaviour, processed meat, insufficient fruit and vegetable consumption, and insufficient fish consumption;
  o Micronutrients/medications: aspirin use, B-carotene, vitamin C, vitamin E, and folate all potentially lower risk;
  o Occupational exposure to organic dust.

More high-quality studies are required to understand risk factors for EAO-CRC, particularly prospective cohort studies on generalizable populations. These include studies that compare risk factors between early-onset and later-onset CRCs, and studies of novel exposures or exposures unique to recent cohorts. 

To pursue these questions, Canadian researchers will utilize data from the Canadian Partnership for Tomorrow’s Health (CanPATH), the largest study of its kind in Canada, involving more than 300,000 Canadians from eight provinces. This consortium of clinical researchers from across Canada will:

- Examine associations of potential risk factors with the development of EAO-CRC;
- Compare the magnitude of risk associated with different exposures between early and later-onset CRC (60+);
- Identify combinations of risk factors that are associated with the highest risk of developing EAO-CRC.

Results from this research endeavour may identify risk factors and combinations that put certain patients at higher risk. These findings will have important implications for the primary prevention and targeted screening of younger populations.

### 2.5. Secondary Prevention: Early Detection

The early detection of CRC in younger populations is another area where improvements are needed. The successful identification of CRC in its earliest stages, when it is most treatable, depends both on patients seeking medical attention for their symptoms and on these being immediately investigated. Routine CRC screening of otherwise healthy individuals is proven to be effective in improving outcomes for people over the age of 50 [23,24,25]. However, in Canada, younger populations are excluded from organized CRC screening programs unless the patient has been designated as being high risk, such as having a first-degree relative who was diagnosed the disease.

Younger patients are often unaware of and delay seeing their primary care provider about symptoms of CRC, which may contribute to the later stage at diagnosis. The “Never Too Young” survey found that 49% of respondents had no knowledge of signs and symptoms of CRC before their diagnosis [16]. A recent U.S. study found that the median time from onset of bleeding to diagnosis of CRC in patients under age 50 was 180 days, with longer duration noted in advanced cancer [26]. Another study of rectal cancer patients showed that those under 50 had symptoms of rectal bleeding for a much longer time before presenting to a primary care provider than did their counterparts over the age of 50; they also were later referred to a specialist. Total time from symptom onset to treatment was 217 days for younger patients compared with 58 days for those over 50. For younger patients, nearly half of this interval was due to a delay in their seeking medical attention—121 days compared with 21 days in the older age group [27].

Primary care providers may not suspect a diagnosis of CRC in a younger patient. The “Never Too Young” survey reported that initially many patients (54%) were misdiagnosed, and that their symptoms were most commonly mistaken for hemorrhoids, anemia, irritable bowel syndrome (IBS), and mental health issues [16].

Once CRC is suspected, screening is required to confirm the diagnosis. Yet, treatment pathways are not always followed. A Canadian study found that only two-thirds of patients received the correct CRC screening test at the appropriate time and only half reported that their family physicians recommended CRC screening [22]. A recent review from the U.S. showed less than 50% adherence to recommended guidelines for age to start and screening interval among people with a CRC family history [28].

In the U.S., colorectal screening guidelines were recently revised based on a comprehensive review of the evidence of benefiting for various age groups. The U.S. Preventive Services Task Force concluded with moderate certainty that screening for CRC in adults aged 45 to 49 years has moderate net benefit, and in May 2021, recommended screening using the fecal immunochemical test (FIT) for this age group [29]. 

### 2.6. Tertiary Prevention: Reducing Metastasis and Recurrence

Several factors must be considered in determining a treatment approach for a younger patient. CRCs in younger people are generally diagnosed at a more advanced stage and are more aggressive in nature. In addition, distinguishing tumour characteristics in EAO-CRC may have implications for treatment choices, including the tumour’s genomic profile, histology and topography.

A study in Alberta [30] found that younger patients:

- Have more lymph nodes examined;
- Are more likely to receive adjuvant chemotherapy in early stages;
- Are more likely to receive radiotherapy in all stages;
- Are more likely to receive multiagent therapies and more lines of therapy in stage IV disease.

Yet, the benefits and harms of this approach for EAO-CRC patients are unknown.

Upon diagnosis, patients with stage III CRC (and some patients at stage II with certain tumour characteristics) are typically assessed in consultation by medical oncology to discuss the potential role for treatment with adjuvant chemotherapy after resection. Rectal cancers are more common in younger patients, and these are often recommended to be treated with neoadjuvant radiation therapy (with or without chemotherapy) to shrink the mass prior to its surgical removal. A multidisciplinary team typically discusses all options prior to treatment. 

Following surgery, adjuvant chemotherapy is often given for early stage (I–III) patients to reduce risk of recurrence by eradicating micrometastatic disease. Since most recurrences occur within the first 3 years of diagnosis, patients are surveyed closely during this period. Approximately 40% of patients diagnosed with Stage IV CRC will develop recurrent metastases and about 25% will have synchronous metastases in other organs. Approximately 25% of these patients will have metastases in the liver only, which may or may not be resectable [20]. 

The steady introduction of new systemic therapies for CRC (both early- and late-stage disease) over the past 2 decades has accounted for meaningful improvements in survival. More recently, first-line targeted therapies such as monoclonal antibodies targeting EGFR or VGFR, in addition to chemotherapy, have been introduced as a treatment regimen for metastatic CRC [31,32]. In addition, specific genetic mutations have been identified that predict response to specific systemic therapies. Primary and metastatic or recurrent cancers are now routinely tested for a slate of biomarkers. It is currently recommended that patients with metastatic CRC undergo biomarker testing for BRAF or RAS mutations, as well as MSI status. Finally, there are several emerging biomarkers, such as circulating tumour DNA, that have the potential to improve the detection of recurrences and predict those patients who may benefit most from adjuvant therapy in early-stage disease [33]. 

Other important considerations concern the broader needs of younger patients. These include:

- Mental health (mood, anxiety, depression, coping challenges, fear of recurrence);
- Fatigue;
- Family and relationship concerns (children, fertility, sexual health, altered family roles, psychological functioning of spouse/partner);
- Employment/school (financial pressures, impact on careers);
- Personal considerations (aesthetic outcome, altered body image);
- Lifestyle (diet, exercise);
- Physical health (anemia, bowel habits, stoma, menopause, perioperative care).

The results of the “Never Too Young” patient survey showed that treatment had a significant impact on patients: 73.1% said that treatment affected their ability to play sports or participate in other forms of recreation, 46.2% said it affected their relationships, 65.4% said it affected intimacy with their partner, a quarter reported fertility issues, three quarters reported concerns with their emotional health, half worried about pain and fatigue, and nearly all (97.5%) feared a recurrence [16]. In addition, a large proportion of patients (76.8%) had to take leave/quit a job or school. Many respondents were at the peak of their career when diagnosed; therefore, this professional interruption may take a significant and long-lasting toll on career goals and financial stability [16]. Finally, there is some evidence that patients are not sufficiently informed on these side effects. For instance, 59.7% of patients did not receive guidance on the sexual or fertility side effects of treatment [16].

## 3. Issues and Actions

Based on the evidence presented, the following issues were identified as being both important for improving outcomes in EAO-CRC and actionable in the short term:

- Increased awareness of EAO-CRC;
- Promotion of earlier detection in younger populations;
- Need to improve the primary care health system in Canada such that every citizen has access to a primary care provider that they can see at least once per year;
- Continuation of research to identify unique risk factor profiles, tumour characteristics and treatment models that can inform tailored EAO-CRC management approaches.

### 3.1. Improve Awareness of Risk Factors and Symptoms

Both the general public and primary care practitioners need to be informed of and reminded about the most common signs and symptoms of CRC in younger age groups; to understand and share information about risk factors (especially family history); and to seek the earliest possible referral for screening. Primary care practitioners need to recognize the importance of taking a good family history. Accurate diagnosis and prompt action are essential.

Educational resources are readily available. For example, the CCRAN website (ccran.org) offers the *Colorectal Cancer Physician Toolkit* (Physician Tool Kit-Poster (ccran.org)), which is designed to be posted in physicians’ offices and distributed to patients. The website of Genetics Education Canada–Knowledge Organization (geneticseducation.ca) offers educational resources, point-of-care tools, information about cancer genomics, and PowerPoint© seminars for patients and healthcare professionals.

### 3.2. Expand Organized Screening Programs

Including younger Canadians in organized CRC screening programs would avoid some of the pitfalls seen in the current approach to symptom recognition and opportunistic screening. A review of the Canadian Task Force on Preventive Health Care’s 2016 recommendations on screening for CRC [34] may be beneficial, based on the emerging evidence of the disease in younger Canadians. 

In the near future, as results of ongoing research into risk factors and tumour profiling become available, a more specific, risk-stratified approach to the screening of younger people can be developed. 

### 3.3. Invest in Research

Much more evidence is needed to build a robust understanding of EAO-CRC [35]. Recommendations for further population-based research directions include:

- A detailed understanding of the trends in histology, topography, initial stage at diagnosis, and mortality among EAO-CRC;
- Detailed analysis of cost-effectiveness outlining the impacts on current screening programs with potential harms and benefits;
- A comprehensive understanding of risk factor profiles that may lead to meaningful recommendations for screening decisions within the 40–49 age group in the absence of wide-spread screening programs;
- An evaluation of the effectiveness of current and novel screening tests or biomarkers specifically in the 40–49 age group.

## 4. Conclusions

This first symposium on EAO-CRC brought together patients, healthcare providers, researchers and policy makers from across Canada and the U.S. to identify key issues and review successes, as well as to hear perspectives on how to save lives by building awareness and the early detection of the disease. Table 2 summarizes key actions from the symposium.

Participants learned about the urgent need to take action in the face of rapidly rising rates of CRC among people under 50, the aggressive nature of the disease, and its distinctive characteristics compared to tumours in older patients, including specific impacts of the disease on the lives of younger patients. Although scientific evidence is presently scarce, several new studies are underway that will help researchers to investigate and connect molecular differences with clinical risk factors. Through the increased understanding of the nature of this disease, new tools can be developed for the earlier detection and treatment of CRC in younger people.

This symposium began the conversation. In the near future, a second symposium will be held, engaging a broader audience, to discuss new evidence and models of care to improve outcomes in EAO-CRC in Canada. 

## Figures and Tables

**Table 1:** EAO-CRC Symposium agenda.

Session	Speakers
Welcome and Introduction	Ms. Filomena Servidio-Italiano, CEO and President, CCRAN Dr. Christopher Lieu, Associate Director for Clinical Research, University of Colorado
The Patient’s Lived Experience: Young Adult Patient Panel Discussion	Facilitator: Ms. Dawn Richards, PhD, President, Five02 Labs and Global Patient Advocate Panel: Mr. Andrew Hare, Colorectal Cancer Survivor Ms. Ana Bettencourt, Colorectal Cancer Patient Ms. Armina Ligaya, Colorectal Cancer Survivor Mr. Bill McGinley, Colorectal Cancer Patient
The Impetus for Change in the U.S.	Ms. Kim Newcomer, Never Too Young Program Manager, Colorectal Cancer Alliance Ms. Andrea Dwyer, University of Colorado Cancer Center & Fight Colorectal Cancer
Exploring the Landscape of Ongoing Research Initiatives in EAO-CRC	Dr. Darren Brenner, Molecular Epidemiologist, University of Calgary
Colorectal Cancer: Why Family History Matters	Dr. June Carroll, Family Physician, Sinai Health System, University of Toronto
Treatment Pathways: Management of Early and Later Stage Disease	Dr. Shady Ashamalla, Head, Colorectal Cancer Surgical Oncology, Sunnybrook Health Sciences Centre, Toronto Dr. Petra Wildgoose, Family Physician, Sunnybrook Health Sciences Centre, Toronto Dr. Sharlene Gill, Medical Oncologist, BC Cancer Agency, Vancouver
The Case for Early Detection	Dr. Clarence Wong, Interim Section Chief, Gastroenterology, Alberta Health Services, Edmonton
Summary and Wrap Up	Ms. Filomena Servidio-Italiano

**Table 2:** Summary of key actions from symposium.

Action	Outcomes	Who
Increase awareness of EAO-CRC	General public and primary care practitioners know the risk factors and symptoms of CRC in people > 50	Primary care practitioners Oncologists Patient support groups Health educators
Promote earlier detection in younger populations	CRC screening programs include ages 45+	Provincial health ministries
Increase number of family physicians in Canada	All Canadians can see a family physician at least once per year	Provincial and federal governments Medical associations and colleges
Support research to identify unique risk factor profiles, tumour characteristics and treatment models	Risk-stratified approaches to managing EAO-CRC	Medical researchers Government and non-government funders
